# Early clinical experience with the new generation Pipeline Vantage flow diverter in the treatment of unruptured saccular aneurysms using short-term dual antiplatelet therapy

**DOI:** 10.1177/15910199231205047

**Published:** 2023-10-05

**Authors:** Katja Döring, Abdallah Aburub, Joachim K Krauss, Josef M Lang, Shadi Al-Afif, Manolis Polemikos, Karin Weissenborn, Gerrit Grosse, Dominik Grieb, Heinrich Lanfermann, Friedrich Götz, Omar Abu-Fares

**Affiliations:** 1 Department of Diagnostic and Interventional Neuroradiology, 9177 Hannover Medical School Hannover, Hannover, Germany; 2 Department of Neurosurgery, 9177 Hannover Medical School, Hannover, Germany; 3 Department of Neurology and Clinical Neurophysiology, 9177 Hannover Medical School, Hannover, Germany; 4 Department of Radiology and Neuroradiology, Sana Kliniken Duisburg, Duisburg, Germany

**Keywords:** Vantage flow diverter, intracranial aneurysm, DAPT

## Abstract

**Purpose:**

The Pipeline Vantage flow diverter with Shield technology (PV) used in this study is a 4th-generation flow diverter (FD) designed to reduce thrombogenicity, promote endothelialization of the implant and increase efficiency in achieving aneurysm closure. In this study, we report the aneurysm occlusion rate, complication rate and clinical outcome with short-term dual antiplatelet therapy (DAPT) in the treatment of unruptured intracranial saccular aneurysms using the PV.

**Methods:**

We retrospectively identified patients treated between September 2021 and January 2023 with the PV and subsequently underwent short-term DAPT for 3 months. Patient and aneurysm characteristics, peri- and post-procedural complications, clinical outcomes and the grade of aneurysm occlusion were documented.

**Results:**

Thirty patients with 32 aneurysms were treated. Successful FD implantation was achieved in all cases (100%). No periprocedural complications were documented. The overall symptomatic complication rate was 10% and the neurologic, treatment-related symptomatic complication rate was 6.6%. Only one symptomatic complication (3.3%) was device-related. Permanent clinical deterioration occurred in 2/30 patients (6.6%), leading to deterioration of the mRS within the first 3 months after treatment. No mortality was documented. The rate of complete aneurysm occlusion after 3 months and after a mean imaging follow-up of 9.9 months was 65.6% and 75%, respectively.

**Conclusion:**

Implantation of the PV for the treatment of saccular intracranial aneurysms achieves a good aneurysm occlusion rate with a low rate of complications. In addition, the use of short-term DAPT after PV implantation appears to be safe.

## Introduction

In the last decade, the flow diverter (FD) emerged as a widely accepted method for the treatment of intracranial aneurysms. The concept of flow diversion is based on the principle of redirecting blood flow along the long axis of the parent artery, away from the aneurysm. This is achieved through endoluminal placement of the tubular implant over the neck of the aneurysm.^1^ Consequently, aneurysm occlusion occurs secondarily to thrombosis after interruption of turbulent flow into the aneurysm sac. The key element of FD stent design is a braided mesh. The porosity of the FD mesh and the pressure gradient between the main vessel and the smaller adjacent vascular branches maintain the flow and patency of the latter, even when they are covered. Non-occlusion or occlusion of the aneurysm and potential complications follow device-specific patterns. The prerequisite for the appropriate case-oriented selection of the FD to be used and correct execution of the procedure is an understanding of the technical features and functions of the different devices.

The first generation of FDs, including FRED (MicroVention), Pipeline (Medtronic), Silk + (Balt Extrusion) and Surpass (Stryker) require 0.027-inch inner diameter (ID) catheters. These implants showed high thrombogenicity, which contributed to the relevant incidence of thromboembolic complications.^2,3^

Ideal characteristics of a FD include optimal pore density and good wall apposition to achieve blood flow stagnation in the aneurysm,^4^ lower overall implant thickness profile to improve endothelial coverage of the parent vessel and aneurysm neck and low thrombogenicity to mitigate ischemic complications.

The Pipeline Vantage flow diverter with shield technology (PV) is a 4th-generation novel FD. PV is composed of either 48 (for 2.50–3.50 mm braids) or 64 (for 4.00–6.00 mm braids) cobalt chromium wires, each featuring platinum on their inner surface in a drawn-filled tube configuration. The 64-wire implant contains additional 16 solid cobalt-chromium wires in order to increase the radial force of the FD. Notably, these wires have a smaller diameter than those found in the previous version of the device, resulting in a reduced overall thickness profile of the implant. This characteristic has the potential to promote endothelial growth over the struts of the FD and thus improve the aneurysm occlusion rates. The phosphorylcholine surface modification of the PV device resulted in similar clinical performance in aneurysm occlusion while improving the safety profile through reduced thrombogenicity of the implant material and potentially promoting early neointimal coverage.^5–7^ Despite its lower thrombogenicity, it is crucial to emphasize that the manufacturer does not recommend using the PV without dual antiplatelet therapy (DAPT).

The purpose of this single centre analysis is to assess safety and efficacy of the new PV for the treatment of intracranial saccular aneurysms.

## Material and methods

### Patient selection

We retrospectively reviewed our database to identify all patients with intracranial saccular aneurysms in the anterior and posterior circulation treated with PV between September 2021 and January 2023. Inclusion criteria for this series were all saccular aneurysms treated primarily with PV implantation only or aneurysm recurrence following prior coiling or clipping. Only patients with unruptured aneurysms or past the acute stage of SAH (>90 days) were included. Fusiform, dissecting and blister-like aneurysms were excluded. Aneurysms treated with other parent vessel implants such as other FDs or stents were also excluded. Demographic data, aneurysm location, clinical and radiological outcome were documented for each patient. Aneurysm and patient characteristics are summarized in Tables 1 and 2.

**Table 1.** Summary of patient and aneurysm characteristics.

Characteristic			
Patients	30		
Sex (m/f)	7/23		
Age (y)	55.0 ± 9.4		mean ± std
mRS before PV implantation	0.0 ± 0.2 (0.0/1.0)		mean ± std (min/max)
mRS at discharge	0.0 ± 0.2 (0.0/1.0)		mean ± std (min/max)
mRS 3 months after PV implantation	0.2 ± 0.6 (0.0/2.0)		mean ± std (min/max)
Aneurysm width (mm)	5.1 ± 2.2 (1.3/9.7)		mean ± std (min/max)
Aneurysm depth (mm)	4.6 ± 2.6 (1.0/11.8)		mean ± std (min/max)
Aneurysm neck (mm)	4.2 ± 1.5 (1.1/7.5)		mean ± std (min/max)
Parent artery diameter min. (mm)	3.3 ± 0.6 (2.1/4.5)		mean ± std (min/max)
Parent artery diameter max. (mm)	4.1 ± 0.7 (2.4/5.3)		mean ± std (min/max)
Parent artery diameter at the level of the aneurysm (mm)	3.8 ± 0.9 (2.0/6.1)		mean ± std (min/max)

Data indicated as mean ± SD (minimum–maximum) or absolute number of patients.

mRS: modified Rankin scale.

**Table 2.** Aneurysm location.

*Anterior circulation*		25 (78.1%)
	*location*	
	Paraophthalmic ICA	16 (50%)
	Pcom	6 (18.8%)
	MCA	2 (6.2%)
	ACA	1 (3.1%)
*Vertebrobasilar circulation*		7 (21.9%)
	*location*	
	SCA	3 (9.4%)
	Basilar tip	1 (3.1%)
	Mibasilar	1 (3.1%)
	Verterbral artery V4-segment	1 (3.1%)
	PICA	1 (3.1%)

Data indicated as absolute number of aneurysms (relative frequency in %). Relative frequency related to the number of aneurysms (*n* = 32).

ACA: anterior cerebral artery; ICA: internal carotid artery; MCA: middle cerebral artery; SCA: superior cerebellar artery; PICA: posterior inferior cerebellar artery; Pcom: posterior communication artery.

### Dual antiplatelet therapy

24 h before the intervention, the patient was informed in detail and written informed consent was obtained. We have implemented a double-loading dose protocol of clopidogrel as a precautionary measure to account for potential non-compliance with the drug in specific individuals. It is worth noting that similar management protocols have been previously published in the cardiological literature, providing a basis for this approach.^8^

Patients received a loading dose of 100 mg acetylsalicylic acid (ASA) and 600 mg clopidogrel the night before the procedure. On the morning of the procedure, a repeat loading dose of 100 mg ASA and 300 mg clopidogrel was given. Starting the day after the procedure, patients received a maintenance regimen of 100 mg ASA and 75 mg clopidogrel. The medication was continued for 3 months and followed by monotherapy of 100 mg ASA 1× daily indefinitely.

### Endovascular procedure

Diagnostic subtraction angiography (DSA) was performed before the intervention in all cases. Based on this, a treatment decision was made by a multidisciplinary neurovascular board.

All procedures were carried out under general anaesthesia via femoral artery access using a Canon Alphenix Sky + biplane digital subtraction angiography system (Canon Medical Systems, Neuss, Germany). Patients were heparinized with a bolus of 5000 IU unfractionated heparin (IVCO Healthcare LLC, Mongolia). Typically, a 6F Neuron Max (Penumbra) was used as the delivery sheath. After placement of the coaxial access system, the sequence of subsequent treatment steps was similar: first, a distal access catheter (DAC) (Navien, Medtronic or Sofia, Microvention) was placed proximal to the aneurysm. Next, a Phenom 0.027″ or 0021″ microcatheter (Medtronic) was advanced into the parent vessel. The FD was then deployed in the landing zone of the parent vessel to cover the aneurysm neck. The endovascular procedure ended with DSA runs in the working and standard projections.

### Radiographic and clinical follow-up

**Figure 1.**
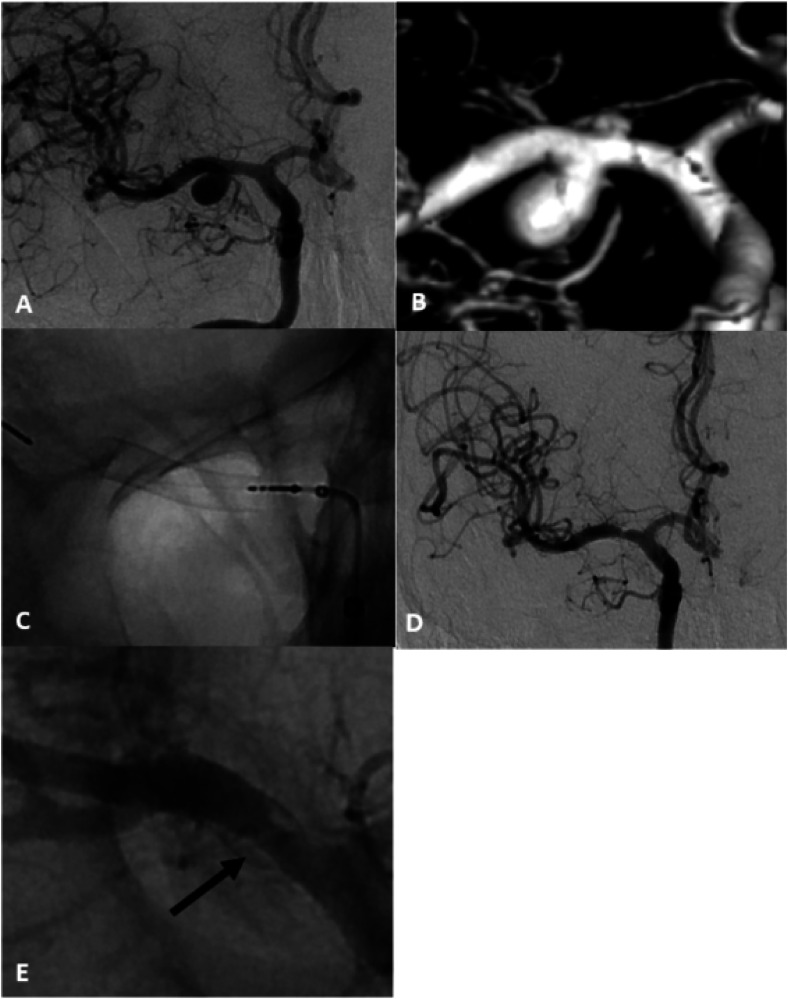
Flow diverter (FD) implantation for the treatment of an unruptured saccular M1-segment side-wall aneurysm of the middle cerebral artery (MCA). Digital subtraction angiography (DSA) (A) and three-dimensional reconstruction of a rotational angiography anterior view (b) show a saccular aneurysm of the right MCA with a maximal diameter of 6 mm. After positioning of a Phenom 21 microcatheter in the MCA distal to the aneurysm a 3 × 12 mm Pipeline Vantage FD was implanted (C). In the control DSA 6 months after the treatment, the aneurysm was completely occluded (D, E). Moderate thickening of the vessel wall with moderate stenosis was noted at the proximal portion of the stent due to fish mouthing (black arrows in E).

Three months after the intervention, the first follow-up was in the form of magnetic resonance imaging (MRI), including fluid-attenuated inversion recovery (FLAIR), time of flight (ToF) and diffusion-weighted imaging (DWI). Further follow-up, that is, frequency and modality, depended on the primary MRI findings. If no signs of a haemorrhagic or ischemic event were visible on MRI, further follow-up (usually MRI) was scheduled 8–12 months after FD implantation and clopidogrel was discontinued (continuation of ASA-monotherapy 100 mg daily). On the other hand, if a haemorrhagic, ischemic event or suspected moderate to severe in-stent stenosis (ISS) were diagnosed, DSA was carried out. If DSA confirmed moderate to severe ISS, DAPT was extended and control MRI was scheduled in three months. Subsequently, DAPT was only discontinued when control MRI showed improvement in morphology (Figure 1).

Clinical evaluation was carried out by neuroradiologist, neurosurgeon or neurologist before the intervention, immediately after the intervention, at discharge and at the follow-up visits. The patient's clinical condition was evaluated using the modified Rankin-Scale (mRS). The degree of aneurysm occlusion was assessed using the Raymond-Roy occlusion rate (RROC).^9^

## Results

### Baseline characteristics of the study population

From September 2021 to January 2023, 30 patients who fulfilled the previously determined inclusion criteria were identified. The mean ± SD age of the patients was 55.7 ± 9.4 years. Of these, 23 patients (76.7%) were female, 7 were male (23.3%). Six patients (20%) received a 48-wire PV and 24 patients (80%) received a 64-wire PV. Two patients who each had two tandem ICA aneurysms were treated with one FD covering both aneurysms.

The 30 patients harboured 32 aneurysms, all of which we treated with PV: 25 aneurysms (78.1%) were located in the anterior circulation and 7 aneurysms (21.9%) were located in the vertebrobasilar territory (Table 2).

The minimum diameter of the parent artery was on average 3.3 ± 0.6 mm (mean ± SD) (range 2.1–4.5 mm), the maximum diameter of the parent artery was 4.1 ± 0.7 mm (range 2.4–5.3 mm). The parent artery diameter at the level of the aneurysm was 3.8 ± 0.9 mm (range 2.0–6.1 mm). The mean width of the aneurysm was 5.1 ± 2.2 mm (range 1.3–9.7 mm), the depth of the aneurysm was 4.6 ± 2.6 mmm (range 1.0–11.8 mm), and that of the aneurysm neck was 4.2 ± 1.5 mm (range 1.1–7.5 mm). Post-interventional follow-up at three months was available for all patients (100%). mRS obtained before the intervention was 0.0 ± 0.2 (0.0/1.0). mRS at discharge was 0.0 ± 0.2 (range 0.0–1.0) and 3 months after PV implantation 0.2 ± 0.6 (range 0.0–2.0).

### Qualities of the next generation Fd: safety and efficiency in achieving aneurysm closure

#### Safety

A total of 30 PV FDs were successfully implanted without adjunctive coiling. In our series, we did not encounter any technical difficulties related to inadequately deployed PV devices, such as structural defects, twisting or incomplete wall apposition. No periprocedural thrombus formation was observed. 7/30 post-interventional complications occurred during follow-up and are described below. Patients did not experience clinical deterioration at discharge. During a mean clinical follow-up period of 11.8 months, two patients experienced clinical deterioration with mRS shift. No mortality was observed (Table 3).

**Table 3.** Summary of complications following PV implantation.

*Sex, age*	*Description*	*mRS shift*	*Aneurysm location*	*PV size*
*Asymptomatic complications*				
Female*, 51*	NIH with moderate ISS due to fish mouthing	No	MCA	3 × 12 mm
Female*, 44*	NIH with severe ISS	No	ICA	4 × 12 mm
Female*, 62*	NIH with severe ISS and thromboembolic event	No	ICA	4 × 16 mm
Female*, 41*	ICA occlusion	No	ICA	4 × 16 mm
*Symptomatic complications*				
Female*, 57*	Lobar intraparenchymal haemorrhage	0 to 2	SCA	3 × 20 mm
Female*, 51*	Basal ganglia infarct	1 to 2	MCA	3 × 14 mm
Male*, 50*	Cerebellar infarct due to cardial thrombus found in left atrium	No	Midbasilar	5 × 40 mm

NIH: neointimal hyperplasia; ISS: in-stent stenosis; ICA: internal carotid artery; MCA: middle cerebral artery; SCA: superior cerebellar artery; mRS: modified Rankin scale.

#### Asymptomatic complications

**Figure 2.**
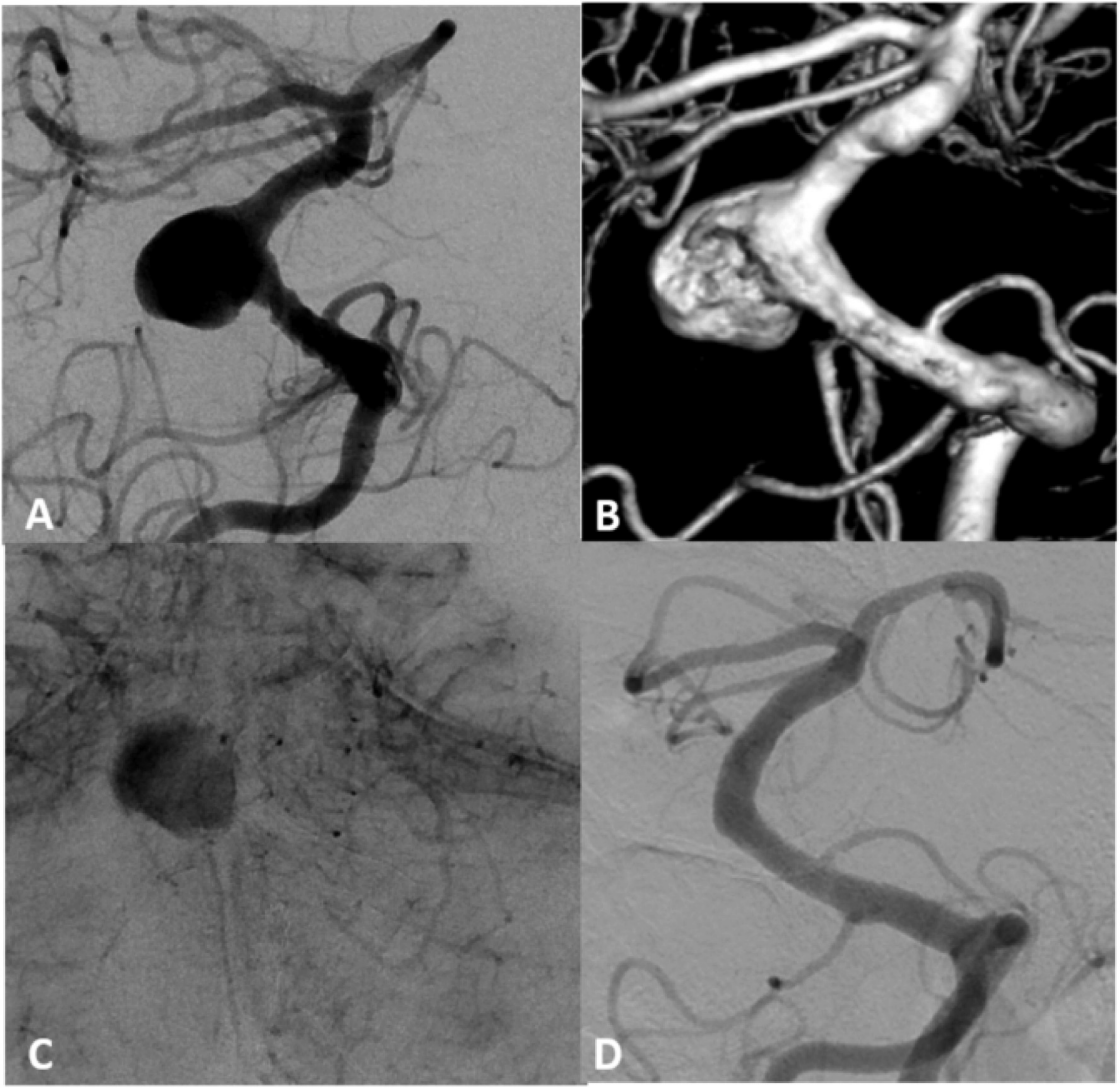
Basilar side-wall aneurysm treated with Pipeline Vantage (PV). Digital subtraction angiography (DSA) (A) and three-dimensional reconstructions of a rotational angiography (B) show a saccular aneurysm located in the mid-basilar segment. Using a Phenom 27 microcatheter, a Pipeline Vantage flow diverter 5 × 40 mm was implanted, extending from the distal basilar artery, covering the aneurysm neck and the anterior inferior cerebellar artery (AICA) proximally. Angiography immediately after the implantation (C) showed stasis of contrast agent within the aneurysm sac. In the follow-up DSA 6 months after treatment shown in (D), the aneurysm was completely occluded with patency of the AICAs bilaterally.

Four clinically asymptomatic adverse events occurred after PV Implantation (13.3%). In three patients, excessive neointimal hyperplasia (NIH) resulting in moderate to severe ISS was detected on the first follow-up MRI 3 months after PV implantation and was later confirmed by DSA. FD deformity was not observed on DSA. These patients showed no neurologic impairment and they underwent salvage therapy in the form of prolongation of DAPT. One patient had asymptomatic occlusion of the ICA, which was also diagnosed on MRI and subsequently confirmed by DSA. No apparent reason could be found for the stent occlusion. The fish-mouthing phenomenon was encountered in one case; this resulted in moderate ISS but without neurologic sequelae. DAPT was prolonged in this case (Figure 2).

#### Symptomatic complications

**Figure 3.**
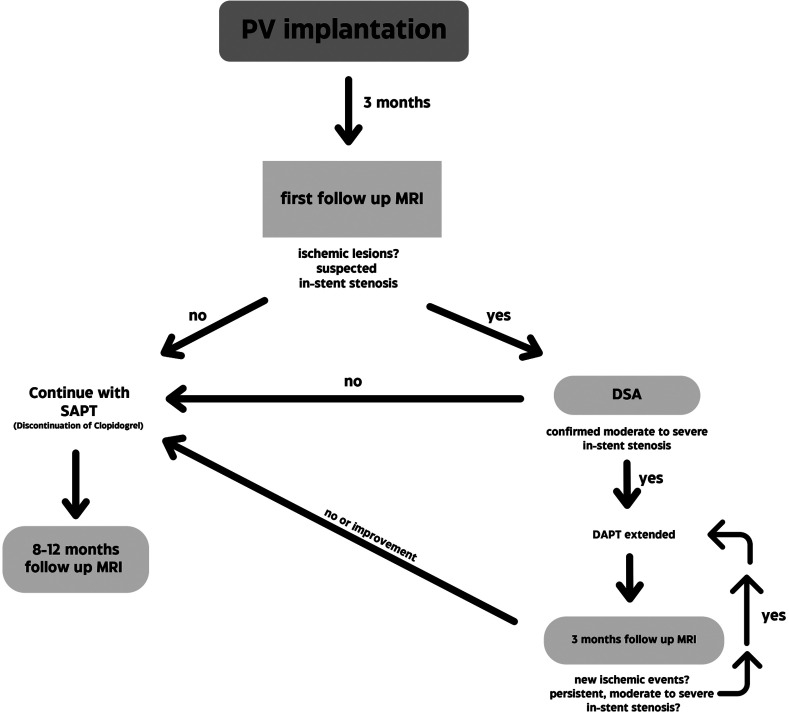
Follow-up management after PV implantation.

About 3/30 symptomatic complications (10%) occurred one to six months after PV implantation: 28 days after the intervention, one patient (57-year-old female) experienced a lobar intraparenchymal haemorrhage, a complication most likely attributable to DAPT since no other cause was found. This resulted in clinical deterioration with a shift in mRS from 0 to 2. Another patient (51-year-old female) suffered a basal ganglia infarction 23 days after PV implantation due to the coverage of middle cerebral artery perforators by the FD. This also resulted in a mRS shift from 1 to 2. Lastly, six months after FD implantation in the basilar artery for the treatment of a basilar side-wall aneurysm, another patient (50-year-old male) suffered a small cerebellar infarction with no mRS shift (Figure 3). This was attributed to a large thrombus found in the left atrium and thus explicitly not associated with FD implantation.

#### Efficacy in achieving aneurysm closure

The first follow-up 3 months after PV implantation was available for all patients (100%) harbouring 32 aneurysms: 21/32 aneurysms (65.6%) showed complete aneurysm occlusion (RROC I), 5/32 aneurysms (15.6%) exhibited a neck remnant (RROC II) and 6/32 aneurysms (18.8%) showed an aneurysm remnant (RROC III). The second imaging follow-up was available for 16/30 (53.3%) patients and for 17/32 (53.1.6%) aneurysms after a mean of 9.9 months after the intervention. 12/17 aneurysms were RROC 1 after the first follow-up and this was confirmed in the second follow-up. 1/17 aneurysms previously assessed as RROC III improved to RROC II, 2/17 aneurysms improved from RROC III to RROC I, 1/17 aneurysms improved from RROC II to RROC I and 1/17 aneurysm remained RROC III after the one year follow-up.

Overall, after a mean imaging follow-up of 9.9 months, the complete aneurysm occlusion, neck remnant and aneurysm remnant was observed in 24/32 (75%), in 5/32 (15.6%) and in 3/32 (9.4%) aneurysms, respectively.

## Discussion

### Qualities of the next generation FD

Based on our retrospective study at a single neurovascular centre, the safety and efficacy of the PV FD in the treatment of saccular intracranial aneurysms is promising.

The desirable characteristics of a FD are optimal pore density, low thrombogenic profile, low implant thickness profile and wall apposition. Accordingly, the PV was designed with (1) thinner wires to generate a lower implant profile and (2) a higher pore density (64 wire implants) than previous models (e.g. Pipeline Flex embolization device). Optimizing the pore density of FDs is important to achieve adequate blood stagnation in the aneurysm, improve occlusion and scaffold formation at the neck of the aneurysm.^10^ This should theoretically translate into improved flow diversion while preserving patency of adjacent perforators and branch vessels as well as high aneurysm occlusion rates, as demonstrated in this study. These two aspects synergistically result in higher tissue coverage and consequently faster occlusion of the aneurysm. With the integration of Shield technology into the PV, the functional advantages of the implant – a reduction in thrombogenicity and early neointimal coverage – evaluated in several preclinical models were realized.^11,12^ This was confirmed in the human blood loop study, which observed a significant reduction in thrombin generation and platelet activation along with reduced deposition of thrombus on the intraluminal surfaces of the PV.

In this single centre study, the PV demonstrates: (a) low thrombogenicity with a low rate of peri- and post-interventional complications; (b) high, partly even overshooting endothelial coverage; (c) a high midterm aneurysm occlusion rate. The rate of technical procedural complications was very low. Previously described hang-up of the FD on the pusher wire was not encountered in any case.

### Efficacy and safety

The rate of complete aneurysm occlusion at three months and after a mean imaging follow-up period of 9.9 months was 65.6% and 75%, respectively. Similar complete aneurysm occlusion rates were previously published for PV and other FDs.^13,14^ In the first multicentre experience with PV, the complete aneurysm occlusion rate reported was 77.9%, although almost 19% of the patients underwent additional coiling. In our study all patients underwent PV implantation with no additional coiling, which could explain the slightly higher aneurysm occlusion rate previously reported.^15^

The Achilles’ heel of FD is thromboembolic or haemorrhagic – mostly due to DAPT – complications.

In our study, in 30 patients harbouring 32 aneurysms treated by the PV, the overall symptomatic complication rate was 10% and the neurologic, treatment-related symptomatic complication rate was 6.6%. All neurologic treatment related complication occurred within the first three months after PV implantation. Of these complications, only one was directly related to the FD (3.3%; infarction due to the coverage of MCA perforators by the FD). Another patient (3.3%) suffered lobar intraparenchymal haemorrhage occurring 28 days after FD implantation, which was likely attributable to DAPT since no other cause was evident. Another delayed device-related complication was complete stent occlusion detected on follow-up imaging, which was asymptomatic. No neurological mortality was observed. Thus, despite the smaller overall cohort and relatively short mean clinical follow-up of 11.8 months, our results are in line with other studies. Charbonnier et al. reported a neurologic complication rate of 18%.^16^ Kallmes et al. reported an overall incidence of a neurologic morbidity rate of 7.4% and intracranial haemorrhage rate of 2.4% in a study of 793 patients with 906 aneurysms.^14^ A recently published multicentre study investigating PV reported an overall symptomatic complication rate 8.2% and neurologic complication rate of 3.3%.^15^

The high sensitivity of MRI to detect ischemic changes on DWI and the ability to depict aneurysms using ToF angiography makes it a suitable imaging tool to assess treatment success after flow diversion.^17,18^ The frequency and type of DWI lesions encountered confirmed the good safety margins of the procedures performed and the devices implanted. A high rate of DWI lesions after FD implantation was previously reported.^19^ In our retrospective analysis, ischemic changes on MRI were detected in two cases (6.6%), without neurologic impairment in one case. Despite these complication rates, 93.33% of patients were functionally without change in clinical outcome three months after PV implantation and 100% were functionally independent (mRS 0–2). Chabonnier et al. reported similar results in their cohort, achieving functional independence (mRS 0–2) in 92%.^16^ In the INTREPED study,^20^ the rate of ischemic events reached 4.5%. In the prospectively conducted DIVERSION observational study^21^ an event-free survival rate of 75.7% was secured, permanent clinically serious events and ischemic events occurred in 5.9% and 14% of the 408 interventions, respectively. Cagnazzo et al. 2019 and Wang et al. 2016 independently established an association between the location of the aneurysm and the incidence of neurological complications.^22,23^ A higher risk was reported for MCA^22^ and posterior circulation aneurysms,^23^ possibly related to a higher rate of perforators at these sites. Similarly, both neurologically symptomatic complications occurred in this study after FD implantation in the posterior circulation and in the MCA.

Meanwhile, it has become established that patients undergoing FD implantation are treated with DAPT before and after the intervention. There is a wide variety of platelet inhibition regimens and duration of treatment when intracranial stenting is carried out and response testing to DAPT is widespread, but its use lacks randomized control trials. Brinjikji reported higher rates of morbidity in patients after Pipeline embolization device placement with platelet testing.^24^ Two studies that performed systematic reviews found similar rates of association between ischemic complications and clopidogrel duration of less than six months.^25,26^ On the other hand, testing of thrombocyte aggregation response could lower the risk of thromboembolic events but not the frequency of major bleeding events.^27^ In our study, midterm clinical follow-up results were available for 29/30 patients (96.7%) at a mean of 11.8 months. Of these patients, no deterioration in mRS was observed three months after PV implantation, which supports the safety of the platelet inhibition regimen used.

The primary desirability of early endothelial coverage of FDs may mitigate thromboembolic events but may also result in ISS due to excessive NIH. In the context of our study, three cases (9.9%) exhibited moderate to severe ISS. This phenomenon did result in thromboembolic events in one case (3.3%) without neurologic sequelae and was compensated by salvage therapy in the form of prolonged DAPT, which was only changed to SAPT when follow-up imaging showed improvement in morphology. One recent study reported only mild NIH in 60 patients treated with PV.^15^ Moderate to severe ISS occurred more frequently and close monitoring of these patients with prolonged DAPT should take place.

Although there is reason to believe that deformity of the FD documented on follow-up studies but not visible in the immediate postinterventional DSA might be associated with ISS, we did not encounter this phenomenon in our ISS cases.

The limitations of this study are the small number of patients and the small number of long-term follow-up results currently available. It is also a retrospective analysis of a single centre study. Some of the results, both imaging and clinical, were subjectively rated by the interventionalist, with no external independent monitoring. Yet the evidence generated during the study encourages further prospective analysis to demonstrate the value proposition of the PV for the treatment of intracranial aneurysms with short-term DAPT.

## Conclusion

The PV FD for the treatment of unruptured intracranial saccular aneurysms was retrospectively analyzed in this clinical study and found to achieve a low complication rate using short-term DAPT without platelet function testing. Moreover, the complete aneurysm occlusion rate on follow-up imaging was promising. Long-term aneurysm occlusion rates and safety remain to be proven by larger prospective studies with a prolonged follow-up.

## Appendix

**Notation**
ASA: acetylsalicylic acid
CT: computer tomography
DAPT: dual antiplatelet therapy
DAC: distal access catheter
DWI: diffusion weighted imaging
DSA: digital subtraction angiography
FD: flow diverter
FLAIR: fluid-attenuated inversion recovery
ICA: internal carotid artery
ISS: in-stent stenosis
MRI: magnetic resonance imaging
mRS: modified Rankin scale
NIH: neointimal hyperplasia
PV: Pipeline Vantage flow diverter
RROC: Raymond-Roy occlusion rate
SAH: subarachnoid hemorrhage
SAPT: single antiplatelet therapy
ToF: time of flight
